# The Bridge Between Real and Ideal: Students Perception on Quality Gap in Reality and Their Educational Expectations

**DOI:** 10.5812/ircmj.14254

**Published:** 2014-08-10

**Authors:** Bahram Nabilou, Davoud Khorasani-Zavareh

**Affiliations:** 1 Social Determinants of Health Research Center, Urmia University of Medical Sciences, Urmia, IR Iran; 2 Road Traffic Injury Research Center, Tabriz University of Medical Sciences, Tabriz, IR Iran; 3 Department of Clinical Science and Education, Karloinksa Institutet, Sodersjukhuset, Stockholm, Sweden

**Keywords:** Student, Perception, Education, Consumer Satisfaction, Medical Students

## Abstract

**Background:**

Studies in higher education indicated that students’ expectation for their educational services are not provided sufficiently, particularly in developing countries that implies on gap between the students perception on current situation and their expectations from educational services.

**Objectives:**

The aim of this study was to determine the gap between student perception and expectations of students in various levels of the undergraduate educational courses at Urmia University of Medical Sciences, Iran.

**Patients and Methods:**

This is a longitudinal study, which was conducted in academic year 2007-2008 at the Urmia University of Medical Sciences. In total, 173 students were selected as sample size, among various courses. SERVQUAL questionnaire was used as instrument. Descriptive statistics following by Friedman and Wilcoxon tests were used to determining significance of quality gap between five dimensions and to evaluate significant gap between student perceptions and their expectations, respectively. Spearman test was also used to determine the relationship between dimensions.

**Results:**

In overall, 80% of educational expectations were not meet; there was a negative gap at all phrases and dimensions and the gap was more negative for educational experts (-1.45 ± 0.89) compared to teachers (-0.97 ± 0.97). The highest gap for teachers was in empathy dimension (-1.11 ± 1.51), while for experts it was in assurance dimension (-1.58 ± 1).

**Conclusions:**

Existences of gap in dimensions indicated that expectations of students are not met and it indicates their dissatisfaction, and thus it is a necessity for improvement in all dimensions.

## 1. Background

Quality improvement in higher education and movement toward knowledge economy is recognized as the base of country development (1, 2). The Quality of a higher education system as well as its monitoring and evaluation are essential to social and economic well-being (3, 4). Students’ perceptions regarding educational environment are considered as an important basis for the quality improvement of their educational environment (5). Moreover, in a university, staff expertise has been acknowledged as the most important source; and without considering their perception, it is difficult to achieve quality improvement (6). Universities as well as other institutions that are affected by market forces, have to put quality, excellence and cost-effectiveness services on their students (7). It is particularly important to extend with more attentions from a quantitative development to a qualitative one both in developed and in developing countries (8). In the educational systems, students’ perceptions are considered as one of the basic improvement routes (9). Higher educational system, like other organizations those providing services should be as a part of the customer services to identify students needs and their expectations as the most important educational clients (8) and ensure that educational service providers will meet the expected quality based on students’ needs (10). International studies on higher education have shown that expectations in higher educational services, did not be provided comprehensively particularly in developing countries (11-13). Studies of the students need and their expectations on educational services in Iran also show that approximately 80% of the student’s expectations are higher than the current educational services (14). To our best knowledge, most research in developing countries as well as Iran were carried out to analyze the gap between the current status and students’ expectations employing cross-sectional design (11-14) and just a few longitudinal studies have been performed to determine the current state of educational services and the expectations of the services comprehensively (15). Students usually do not have a complete understanding of the features and facilities of universities at the time of entering to educational environment, while the expectations of students from the university are largely determined before entrance, which highlights the importance of the longitudinal studies. Moreover, inclusion of both faculty members and educational experts’ by means of student perception can be explained in more details and shows the gap between reality and students’ expectation simultaneously.

## 2. Objectives

Accordingly, this study was designed to determine the gap between reality and expectations of students in various levels of the educational courses that were provided by faculty members and educational experts at Urmia University of Medical Sciences (UMSU), employing longitudinal design.

## 3. Patients and Methods

This is a longitudinal study that was conducted from October 2007-April 2008 as academic year at UMSU in West Azerbaijan Province, Iran. It has four public universities and three private universities and almost all students come from Iran. UMSU as the research unit has four schools including Medicine, Nursing and Midwifery, Health and Paramedics. During study period, there were 616 new students and the sample size was calculate as 173 including 80 paramedics’ students, 75 students studying at the department of Health, 35 medical students, 25 nursing students and 25 midwifery students, respectively.

### 3.1. Sample Size and Sampling Procedure

In total, there were 2100 students at university of which 616 were new coming, as sampling frame. The inclusion criteria were students those were at first semester, those recently entered at university, and those were undergraduate level. Based on the previous study (14) negative gap frequency of the students was considered as % 80 and employing finite formula the sample size was calculated as 173 (P = 0.8, d = 0.05% and z = 1.96). Among new coming students, there were three courses of associate degrees, three course of bachelor's degree, and one course of medical doctoral degree. Of these students, 50 were at paramedics’ school, 50 were studying in the department of Health, 38 were nursing and midwifery and 35 were medical students, respectively. Finally, authors find out 141 completed questionnaires in first and second semester among undergraduate students. The sampling method was according to cluster sampling with regard to distribution of students in each school and course.

### 3.2. Data Collection Tool

Data gathering tool was SERVQUAL questionnaire that have been developed with regard to five dimensions of quality of service by Berry et al. (16). This questionnaire has five dimensions including:

1. Physical and tangible dimension that means sense of physical space of services, including facilities, equipment, personnel, and communication channels;
2. Reliability of service by means of the ability to serve as a safe and reliable service;
3. Responsiveness that is employees' willingness to cooperate and assist students;
4. Assurance that show an organizations' ability to instil a sense of competence and confidence to the customers; and
5. Empathy that is special deal with the students based on their mood.

In this study, students are regarded as customers and educational part of university as organization. The SERVQUAL questionnaire has been used in different countries in various fields including in healthcare services, banking, customer services and the other fields (17-19). In this study we relayed on its Persian version that has been used in two previous studies (14-20). Questionnaire has two main parts. The first part included personal and educational questions and questions regarding age, sex, residential address, educational level as well as educational background at universities. The second part included 22 questions covering the expectations and perceptions about educational services, which were designed in Likert scale, ranged from 1-5. To ensure the validity of content in the education sector; it was approved by experts as well as agreement in an Expert panel. The reliability of the questionnaire was assessed by Cronbach's alpha coefficient that was 0.928 for faculty members and 0.923 for educational experts, respectively.

### 3.3. Data Collection Procedure

Data collection is carried out in two stages. The first stage was related to the students’ expectations of educational services in relation to faculty members and educational experts in the five dimensions covering the quality of service in October 2007. In order to do that, students were asked about their expectations of educational services by 22 questions in five dimensions by choosing one of the items ranged from Very important, Important, A little important, Less important and Very less important. After one semester, the second stage was performed on April 2008 in order to determine their perception about educational services. This part was about students' perception on five dimensions covering quality of service about how to educate, how to deal with faculty member and staff, facilities. At this stage, the students were asked to give their point regarding the current state of educational services by 22 questions in five dimensions ranged from Excellent, Good, Medium, Bad and Very bad. In every stage, the least and the most scores were ranged from 1 to 5. The scores of questions added up in each dimension and the result was divided by the number of the questions of same dimension, so perception and expectation scores in each dimension ranged between 1 and 5. To calculate the overall score for quality of educational services, assigned scores to all questions were added together and was divided to 22 (number of questions) and then the overall quality score ranged from 1 to 5. Quality gap of educational services obtained by subtracting scores of understanding level and the expected level of service perceived. Positive scores of quality gap indicate that educational services is more than the expectations of the students and a negative score of quality gap indicates that it does not meet expectations. Accordingly; the gap score of Zero indicates that the current status of students met expectations of the services and there is no gap between them. Finlay, by comparing two sets of data collected from a constant group in two periods of times, the gap between expectations and the desired status of each dimension were analyzed. After reviewing the completed questionnaire, 17 cases (10%) were removed due to moving of respondents to other universities in the second round and 15 (8.7%) were removed due to incomplete questionnaire (Response rate = 81.5%). Permission for the study was obtained from the Ethics Committee in UMSU (21).

### 3.4. Data Treatment

Both descriptive and analytical statistics were used in this study. Frequency analysis was used to describe the student characteristics and educational background; and to describe quality gap in five dimensions of educational services by means of their gaps. Analytical tests were used to examine quality gap between five dimensions of educational services. Accordingly, Friedman test was used to in order to examine differences in student perception in quality of five dimensions of educational services. To examine the significant gap between perception and expectation, Wilcoxon test was employed. Spearman's test was used to determine correlation and the relationship between five dimensions. SPSS version 13.00 (SPSS Inc. Chicago, IL, USA) was used for data analysis.

## 4. Results

Table 1 presents students’ characteristics at UMSU during study period. Their ages ranged from 18 and 31 years, with a mean of 21 ± 2.1 and median of 20. In general, the lowest negative gap by means of number among 22 items were about the faculty members’ and staff’ item of " Neat and professional appearance" following by " Recording students’ educational documents without mistake ", which were 40.4% and 41.8% of the faculty members and 53.9% and 56.7% in educational experts, respectively. The highest negative gap among 22 questions was” the faculty members accessibility when students need them” with 68.1%, and "to understand the students' educational needs” with 67.4%; and about the educational experts “to understand the educational needs of students "with 78.7% in empathy.

**Table 1.** Students’ Characteristics at Urmia University of Medical Sciences, October 2007-April 2008^a^

Student Characteristics	Results
**Gender**	
Male	57 (40.4)
Female	84 (59.6)
**EducationalLevel**	
Associated degree	83 (58.9)
Bachelor degree	31 (21.9)
Medical doctoral	27 (19.1)
**Course**	
Health	45 (40.5)
Paramedic	38 (18.4)
Medicine	27 (19.1)
Nursing	16 (11.4)
Midwifery	15 (10.6)

^a^Data are presented a No. (%).

Furthermore, the lowest negative gap were in item of "Neat and professional appearance “and” Recording students’ educational documents without mistake" that they were for faculty members 0.42 and 0.23; and for educational experts were 0.63 and 0.67, respectively. In terms of severity, the highest negative gap for faculty members was about "the teachers accessibility when students need them", with a score of 1.23 and "to understand the educational needs of students "with a score of 1.25 and about the educational experts was for “providing service promptly and fast” and "to understand the emotions and values of the students” with a score of 1.82 that was related to empathy dimension. Table 2 presents student perception in five dimensions of service quality in UMSU. In overall, only a small fraction of students’ expectations were met and they believe that the quality of educational services was not based on their expectations and there is a very high negative gap between their expectation and the current situation. Focusing on faculty members’, there was a big negative gap in high proportion of students for empathy, while less gap were identified between assurance and reliability. Focusing on educational experts, there were a negative gap in high proportion of students for assurance and less percent of students determines it for physical and tangibles following by reliability.

**Table 2.** Distribution and Quality gap in Five Dimensional Services in Urmia University of Medical Sciences Based on Students’ Perception in October 2007-April 2008^a^

Quality Dimensions	Lack of Gap	Positive Gap	Negative Gap
**Physical and tangible**			
Teachers	15 (10.6)	20 (14.2)	106 (75.2)
Educational experts	6 (4.2)	17 (12.1)	118 (83.7)
**Reliability**			
Teachers	9 (6.4)	29 (20.5)	103 (73)
Educational experts	8 (5.7)	13 (9.8)	121 (86.5)
**Responsiveness**			
Teachers	13 (9.2)	20 (14.2)	108 (76.6)
Educational experts	7 (5)	11 (7.8)	123 (87.2)
**Assurance**			
Teachers	13 (9.2)	25 (17.7)	103 (73)
Educational experts	4 (2.8)	11 (7.8)	126 (89.4)
**Empathy**			
Teachers	15 (10.6)	16 (11.3)	110 (78)
Educational experts	6 (4.2)	11 (7.8)	124 (88)
**Total**			
Teachers	2 (1.4)	18 (12.8)	121 (85.8)
Educational experts	2 (1.4)	8 (5.7)	131 (93)

^a^Data are Presented as No. (%).

Table 3 presents the mean of expected and perception scores of educational services and its gap among students in UMSU. According to Wilcoxon test, the difference between expectations and their understanding in each of five dimensions of service quality was significant. In overall, the average gap between the desired and current educational services were negative; of which faculty members identified the lowest gap for reliability, while educational experts identified it for physical and tangible dimension. Moreover, the highest gap for faculty members was in the empathy, while for educational experts it was in assurance. Freedman test also indicated that the gap among dimensions were significant (P < 0.001).

**Table 3.** Mean Score of the Students’ Perceptions, Expectations and Service Gap in SERVQUAL Dimensions at Urmia University of Medical Sciences in October 2007-April 2008 ^a^

	Expectation	Perception	Gap
**Physical and tangible**			
Teachers	4.38 ± 0.62	3.49 ± 0.88	-0.89 ± 1
Educational experts	4.11 ± 0.62	3.02±.79	-1.09 ± 0.96
**Reliability**			
Teachers	4.20 ± 0.66	3.37 ± 0.91	-0.83 ± 1
Educational experts	4.27 ± 0.69	2.77 ± 0.96	-1.5 ± 1.1
**Responsiveness**			
Teachers	4.16 ± 0.8	3.07±1.04	-1.09 ± 1.28
Educational experts	4.06 ± 0.83	2.58 ± 1	-1.48 ± 1.2
**Assurance**			
Teachers	4.48 ± 0.56	3.52 ± 1	-0.96 ± 1.1
Educational experts	4.1 ± 0.71	2.52 ± 0.92	-1.58 ± 1
**Empathy**			
Teachers	4.1 ± 1.03	2.99 ± 0.98	-1.11 ± 1.51
Educational experts	3.8 ± 0.84	2.33 ± 0.91	-1.47 ± 1.16
**Total**			
Teachers	4.25 ± 0.59	3.28 ± 0.83	-0.97 ± 0.97
Educational experts	4.07 ± 0.59	2.64 ± 0.77	-1.45 ± 0.891

^a^ Employing Wilcoxon Test, P is significant for all value and is less than 0.001.

Table 4 presents correlation and relationship of dimensions in service quality according to students’ perception in UMSU. Accordingly, correlation in all values was positive and the differences between components were significant (P < 0.001). Spearman test showed that there is a high correlation and positive relationship between the dimensions of service quality and overall results. The highest correlation in faculty members ‘ and experts’ groups was about responsiveness and reliability dimensions, which shows high confidence of students against faculty members’ and experts’ accountability and vice versa. Correlation between empathy and responsiveness also was high, by means of proper accountability by faculty members and experts affected due to the responses, and vice versa.

**Table 4.** Correlation Between Educational Services Quality by Faculty Members and Educational Experts Based on Students Perceptions at Urmia University of Medical Sciences in October 2006-April 2007^a,b^

Service Dimensions	1	2	3	4	5
**Physical and tangible**					
Teachers	1				
Educational experts	1				
**Reliability**					
Teachers	0.567	1			
Educational experts	0.573	1			
**Responsiveness**					
Teachers	0.532	0.782	1		
Educational experts	0.526	0.768	1		
**Assurance**					
Teachers	0.434	0.619	0.676	1	
Educational experts	0.331	0.581	0.625	1	
**Empathy**					
Teachers	0.500	0.622	0.748	0.689	1
Educational experts	0.413	0.572	0.719	0.656	1

^a^n = 141.

^b^ For all Values Correlation is significant at the 0.01 level (2-tailed).

## 5. Discussion

This study is the first in its kind in Iran, by means of its difference from most other studies employing a longitudinal design for two main educational groups of service providers including faculty members and educational experts, from students’ perception for its quality of educational services. Findings indicated that around eight out of tem students’ expectation from educational services didn’t meet and there was a negative gap at all items in all five dimensions of service quality. Moreover, there were more negative gap for educational experts compare to faculty members that were as major finding of this study. This gap in all dimensions indicates a lake of students’ expectations, which imply students’ non-satisfaction, and thus a need to quality improvement in all aspects of educational services. Finding from this study shows that there’s a negative gap among five qualitative dimensions of educational services by students and imply that current educational services did not meet in any range of student expectations in all dimensions. Focusing on faculty members, most of the students had negative gap in empathy and few students had negative gap on reliability and assurance. This comparison for educational experts shows that among most of students, there is a negative gap in reliability and less had negative gap in physical and tangible dimension. Moreover, the highest negative gap among faculty members was for empathy that shows that they might be uninterested to listen to their students and use them less in their activities and students’ access to masters are less at the time of need or don’t accept students’ ideas and views. It seems that the high volume of administrative tasks and increasing number of students in recent years in training and a lack of faculty members’ enough experiences and skills, it make them to not have enough time to express empathy and listen to the students and understand them. Focusing on educational experts, the highest gap was in assurance which may imply that educational experts are not skilful and they require better action and based on a plan or guidance. It seems that educational experts may are not well experienced to provide a sense of trust and confidence and may not have sufficient knowledge and skills in dealing with students. It is also might be because that the educational experts do not behave respectfully that causes sense of un-trust among students. Study in two groups of teachers and educational experts in Sanandaj University have determined a whole negative gap, which was more prominent among experts compared with faculty members that are in line with our findings in this study (15). Moreover, in our study, the highest negative gap for educational experts was about responsiveness and for faculty members was for assurance. It is also important to note that different studies conducted at Medical Universities in Zahedan, Hormozgan, Tehran (14, 20, 22), Azad University (23-25) and Tabriz educational enters (25), which all reported a negative gap among students’ expectations and current satiation in Iran. It can imply that the current educational plan is not based on students’ expectations in most Iranian universities and educational plan may needs to be revised employing students point of views. Findings in Hormozgan (20) and Zahedan (17) Universities of Medical Sciences are also in line with our results, which show there is a negative gap between five dimensions of quality that the highest negative gap is responsiveness, while assurance and Reliability has the lowest negative gap. This may imply that small universities in small cities usually suffer from responsiveness of the authority, which might be because of availability of educational requirements and instruments. In Zahedan University of Medical Sciences, more than 80% of students determined that in overall educational services were weaker than their expectations. However, finding in Tehran University of Medical Sciences shows that the highest negative gap was among empathy and assurance (22). This may imply that Tehran as the Capital city of Iran and the biggest one suffer from empathy that may indicate as a result of more industrialization. Findings of Azad University of Khorskan showed that the highest negative gap was for responsiveness (23). Moreover, in another study in whole branches of Azad University, the highest gape was for assurance and the lowest for Responsiveness (24). A third study in Azad University of Mazandaran showed the highest gape for reliability and the lowest for assurance (25). The results of Tabriz educational centres also showed that the highest negative gap was for empathy (26). On the other hand, studies in Cyprus, Scotland, Pakistan, Australia and Malaysia showed a whole negative gap (27-31). In a study in Cyprus, Assurance and Tangibles had the highest and lowest negative quality gap by means of respectively (27). Research of informational systems at Western Washington University in United States also determined a negative gap but no statistical difference in all dimensions (31). Different results in different studies show that students in different universities have different understandings about quality and its dimensions. This implies that most universities need improving plans that should be based on students’ point of view. Gap existence in the studies was common in all universities that may represent similar conditions of students’ non-satisfactions of service quality in universities. However, the situation is in different ways and different levels for collages. This also may indicate that students have unrealistic position but since they are as customers of the higher education system, their expectation should be considered. 

### 5.1. Strength and Limitation of Study

The limitation of this study was to its focus on just undergraduate students. It is because at the time of study period, there were only a few postgraduate courses, which mainly were at the hospitals and they were worked as residency. Moreover, it was difficult to access postgraduate students at hospitals due to their workload. Additional limitation of the study was its attrition rate, due to the nature of the longitudinal study, however its rate was quite low. The strength of study was to its design as a longitudinal approach and its focus on both students as well as two groups of educational services providers including faculty members and educational experts. This longitudinal approach with focus on two groups of educational services providers including faculty and educational experts indicated that, first, students do not meet their expectations and, second, their empathy and assurance dimension requires more attention. The authors recommend further investigation on background variables and their relationship with five dimensions.
